# Clinical and Radiological Outcomes of Extracorporeal Shock Wave Therapy in Early-Stage Femoral Head Osteonecrosis

**DOI:** 10.1155/2018/7410246

**Published:** 2018-08-19

**Authors:** Abdulrahman D. Algarni, Hassan M. Al Moallem

**Affiliations:** ^1^Department of Orthopedic Surgery, King Saud University, Riyadh, Saudi Arabia; ^2^Department of Surgery, Prince Mohammed Bin Abdulaziz Hospital, Riyadh, Saudi Arabia

## Abstract

**Objective:**

Femoral head osteonecrosis is a progressive clinical condition with significant morbidity and long-term disability. Several treatment modalities including both surgical and nonsurgical options have been used with variable levels of success. High-energy extracorporeal shock wave therapy is a nonoperative treatment option that has been described for early-stage disease. We aimed to assess the functional and radiological outcomes of extracorporeal shockwave therapy (ESWT) in the treatment of osteonecrosis of the femoral head (ONFH).

**Methods:**

Thirty-three hips of 21 patients were included in this study. Adult patients with ONFH of any etiology and in the precollapse stage were included. Clinical (visual analogue scale [VAS] and Harris hip score [HHS]) and radiological (plain radiographs and magnetic resonance imaging [MRI]) evaluations were performed before and after intervention. We used 3000–4500 pulses in a single session performed under general anesthesia.

**Results:**

At an average of 8 months after ESWT, pain scores and HHS were significantly improved compared with the preintervention scores (p<0.001). The overall clinical outcomes were improved in 21 hips (63.3%), unchanged in 5 hips (15.15%), and worsened in 7 hips (21.2%). A trend toward a decrease in the size of the ONFH was observed although not of clinical significance (p=0.235). MRI revealed significant resolution of bone marrow edema (p<0.003). Regression was observed in 9 lesions (42.9%) and progression in 1 lesion (4.7%); no change was observed in the remaining 23 lesions (52.4%).

**Conclusion:**

ESWT is a viable noninvasive treatment option for early-stage ONFH. It significantly improves clinical outcomes and may halt or delay the radiographic progression of the disease in the precollapse stage.

## 1. Introduction

Osteonecrosis of the femoral head (ONFH) was originally described in 1925 as an ischemic necrosis of the hip area [1]. ONFH can affect any joint but most commonly occurs in the hip joint. Its pathology is poorly understood; however, it is known to decrease blood flow to the femoral head leading to cellular death, fractures, and collapse of the articular surface [2, 3].

ONFH is a multifactorial disease with different etiologies ranging from genetic to idiopathic to certain risk factors such as trauma, hematological disorders, and steroid intake [4]. Such pathology is frequently observed in relatively young adults, and most of the untreated patients with ONFH progress to total hip arthroplasty (THA) with a collapse rate of 67% and 85% in asymptomatic and symptomatic patients, respectively [5].

Although the optimal management protocol for patients in the precollapse stage remains unestablished, early intervention before collapse is critical for a successful outcome [1, 5]. High-energy extracorporeal shock wave therapy (ESWT) is a noninvasive procedure that has been successfully used for the last 20 years to manage various orthopedic conditions [6]. ESWT is a sound wave with high pressure and velocity that forms precipitation on the interface between soft tissue and bone [3]. The positive effect of ESWT has been attributed to the reflection and precipitation of shock waves. Multiple studies have demonstrated the therapeutic potential of ESWT in ONFH [7, 8].

In this study, we aimed to assess the short-term clinical and radiological outcomes of ESWT in patients with early-stage ONFH.

## 2. Patients and Methods

The institutional review board of our hospital approved this study. Between September 2006 and September 2011, all patients who presented to our institution and met the inclusion criteria were enrolled in this study. Each patient provided informed consent before participating in the study. Adult patients with ONFH due to any etiology in the precollapse stage, grades I or II according to the Association Research Circulation Osseous (ARCO) classification system, were included in this study. Immunocompromised patients and patients with a history of infection were excluded. The patient demographics are summarized in Table 1.

The evaluation parameters before and after intervention included clinical assessment of pain with a visual analog scale (VAS), where 0 indicated no pain and 10 indicated severe pain, and assessment of function, activity, and motion of the hip using Harris hip score (HHS). Anteroposterior and lateral plain radiographs were obtained before ESWT; at 3, 6, and 12 months after ESWT; and once a year subsequently. Plain radiographs of the hip were used to assess the size of the lesion, the extent of subchondral bone collapse, and the presence of degenerative changes in the hip joint. Magnetic resonance imaging (MRI) was performed before ESWT, at 6 and 12 months after ESWT, and then once a year. These images were used to evaluate bone marrow edema, the size of the lesion, femoral head congruency, the presence of a crescent sign, and degenerative changes in the hip joint. Plain radiographs and MRI images were evaluated by a senior musculoskeletal radiologist who was blinded to the nature of the treatment.

The clinical outcome was defined as “improved” if the patient had ≥50% improvement in VAS and HHS scores, “unchanged” if the patient had <50% improvement in VAS and HHS scores, and “worse” if the patient experienced more severe hip pain and had further restricted hip function than that before intervention [9].

### 2.1. Treatment Protocol

We used the shock wave device OssaTron (HMT High Medical Technologies AG, Switzerland) with a therapy head capable of 350° rotation. After preparing the therapy apparatus and fixing the limb of the affected hip in adduction and internal rotation, ONFH was marked using fluoroscopy in 2–3 points depending on the size of the lesion. We used 3000–4500 pulses (1500 pulses of shock waves for each point); frequency was set at 4/s at 26 kV in a single session, performed with the patient under general anesthesia. The femoral artery was palpated before and after treatment, and lidocaine gel was applied to the skin to maximize shock wave penetration. Local complications such as formation of hematoma, petechial hemorrhage, swelling, deep vein thrombosis, and superficial infection were all recorded.

After the shock wave treatment, the patients were instructed to walk on crutches with non-weight-bearing on the affected extremity for 6 weeks. Follow-up at the outpatient department was performed at 6 weeks, once every 3 months, and then once a year for a minimum of 2 years.

Statistical analysis was performed with SPSS statistical software version 22 (IBM Corp., Armonk, NY, USA). The Student's t-test was used to compare the pre- and postintervention values of VAS and HHS scores. The outcome end points were clinical improvement and conversion to THA. Statistical significance was based on 2-sided p values <0.05.

## 3. Results

Twenty-three patients met our inclusion criteria; of those, 1 patient was excluded owing to inadequate documentation, and 1 patient was lost to follow-up. A total of 21 patients comprising 33 hips with ONFH were included. The average follow-up was 5 years (range, 2–9 years).

At an average of 8 months after ESWT, pain scores and HHS were significantly improved compared with the preintervention scores (p<0.001). The values of VAS and HHS before and after ESWT are summarized in Table 2. The overall clinical outcomes after ESWT are summarized in Table 3. Improvement was seen in 21 hips (63.3%), whereas no change was seen in 5 hips (15.15%) and 7 hips (21.2%) worsened. At the most recent follow-up, 4 hips were converted to THA as their symptoms had deteriorated at an average of 3.7 years after ESWT (range, 2–8 years).

**Figure 1:**
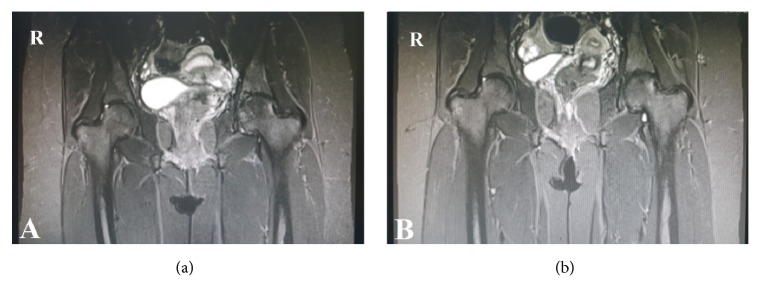
T1-weighted MRI image showing grade II ONFH of the left hip before ESWT (a). The lesion regressed to grade I at 2-year follow-up after ESWT (b).

The changes on plain radiographs and MRIs before and after ESWT are summarized in Table 4. Although a trend toward a decrease in the size of the ONFH after ESWT compared with the preintervention size was seen, it was not clinically significant (p=0.23). On plain radiographs, 2 lesions (6.06%) showed improvement and 5 lesions (15.1%) showed progression. No change was seen in the remaining 26 lesions (78.78%). MRI showed regression of 9 lesions (42.9%; 7 of grade II and 2 of grade I) and progression of 1 lesion (4.7%; of grade II to III). The remaining 23 lesions (52.4%) showed no change (Figure 1). MRI revealed a significant overall improvement of bone marrow edema (p<0.003) after ESWT compared with the preintervention images. Bone marrow edema decreased among patients from grades 3–4 to 2–1 after ESWT (grade 0 stands for no bone marrow edema, 1 for perinecrotic bone marrow edema, 2 for bone marrow edema extended into the femoral head, 3 for bone marrow edema extended into the neck of the femur, and 4 for bone marrow edema extended into the inter-trochanteric region) [10].

There was no difference in clinical or radiological outcomes according to the etiology of the ONFH. No major complications were seen. Nine hips (27.3%) developed device-related petechial hemorrhage in the groin at the site of ESWT application that spontaneously resolved in few days.

## 4. Discussion

Management of ONFH depends on the stage of the disease. For an early-stage disease (grade I and II), before subchondral collapse, both conservative and surgical treatment options have been described. Among the different femoral head preservation procedures (core decompression, muscle-pedicle grafting, and derotational osteotomy), core decompression with bone grafting is considered the gold standard surgical option [11]. Similarly, various conservative treatment modalities have been proposed in the literature as a standalone treatment or in combination with each other [12] These include restriction of weight-bearing, pharmacologic agents (bisphosphonates, low-molecular-weight heparin, statins, and prostaglandins), and biophysical modalities (hyperbaric oxygen, ESWT, and pulsed electromagnetic field therapy) [13]. Lack of sufficient evidence precludes strongly recommending a certain type of conservative treatment in early-stage ONFH; however, the existing evidence favors ESWT [1, 12]. Weight-bearing restriction alone is insufficient; evidence recommending pharmacologic agents is limited, and they have potential side effects. Remaining biophysical modalities (hyperbaric oxygen and pulsed electromagnetic field therapy) have both limited availability and high cost [11–13]. Conservative treatment may be a major focus for orthopedic studies in the future to determine which modality carries the best cost-benefit ratio to establish a standard of care for the treatment of ONFH. Regardless of the selected treatment method, the aim is to improve quality of life, prevent femoral head collapse, improve the speed and quality of repair at the molecular level, and avoid surgical intervention, if possible. The results of this study show that ESWT could help and improve the clinical outcome and possibly cease or retard the progression of the disease eventually.

ESWT is an effective therapeutic option for ONFH, particularly for early-stage disease [7, 8, 14–19]. At grade III and beyond, it is a less viable treatment option [12]. Only few studies with an objective similar to our study exist in the literature [14–17]. Most existing studies highlight the improvement in pain and function, while radiological results varied among these studies. The clinical improvement seen in our study is similar to that reported in previous studies [14, 15]. Although the findings of this study are in agreement with those of other studies with regard to lesion regression after ESWT as seen on MRI, conflicting evidence also exists, mandating further research in this regard [18, 19]. In fact, most studies, including our study, included a small patient population and had limitations such as being uncontrolled and not blinded, which precludes drawing a definitive conclusion. However, evidence indicates that ESWT can resolve bone marrow edema and at least halt disease progression. In the present study, patients had similar clinical and radiographic outcomes regardless of the etiology of ONFH. This finding is consistent with reports by other authors [14, 20].

The exact mechanism of action of ESWT remains unknown. Experimental animal studies demonstrated a significant increase in the ingrowth of neovascularization associated with increased expressions of angiogenic growth indicators in tendon, bone, and tendon-bone interface, which might play a role in the improvement of blood supply and healing, which in turn can improve subchondral bone remodeling and prevent femoral head collapse [21–23]. A close relationship between the decrease of substance P release and consecutive pain reduction after shock wave treatment has also been reported [24–26]. Based on these findings, we can argue that the enhanced angiogenic and osteogenic mechanisms together with hyperstimulation analgesia might play a role in the improvement of blood supply to the femoral head and promotion of bone regeneration.

Reports suggest that although ESWT is an effective standalone treatment modality, combining it with other conservative modalities does not improve its curative benefit [18, 27]. Wang et al. [28] compared the outcomes of ESWT alone and in combination with alendronate in 2 randomly distributed groups. The authors found that clinical outcomes were improved in 83% and 77% of the patients in the ESWT alone group and the combined group, respectively. They concluded that ESWT is an effective treatment with or without the concurrent use of alendronate. Similarly, Hsu et al. [29] have compared the outcomes of ESWT alone and as part of a cocktail treatment consisting of ESWT, hyperbaric oxygen, and alendronate. The authors reported improvement in 79.2% of the patients in the ESWT group, without a significant difference between both groups. In the current study, the therapeutic effect of ESWT, as a conservative treatment, is comparable to that reported following femoral head core decompression for early-stage ONFH. Sen et al. [30] reported a success rate of 63.5% in their series of femoral head core decompression for ONFH. Furthermore, Wang et al. [16] reported even better long-term outcomes with ESWT than with core decompression and bone grafting for early-stage ONFH.

This study has limitations inherent to a retrospective cohort study. Other limitations include the small sample size and the lack of a control group for comparison.

## 5. Conclusion

ESWT is a safe, noninvasive treatment method for ONFH. It helped to improve clinical outcomes in our patients and may delay the radiographic progression in early-stage disease and therefore mitigate the need for surgery. It is a suitable treatment option to be considered, particularly in the precollapse stage of the disease. Further studies are required to validate the long-term and sustained effects of ESWT in ONFH.

## Figures and Tables

**Table 1:** Patient demographic characteristics.

Variable	Values
Patients/hips (no.)	21 (33)
Age*∗* (years)	37.5 ± 4.8 (21-54)
Male/female (no. of patients)	9/12
Right/left (no. of hips)	14/19
Bilateral disease (no. of patients)	12
Duration of symptoms*∗* (months)	6 ± 3 (3-9)
ARCO grade (no. of patients/hips)	
** **Grade-I	4/5
** **Grade-II	17/28
Medical history (no. of patients/hips)	
** **Sickle cell disease	11/18
** **Systemic lupus erythematosus	2/3
** **Steroid intake	3/3
** **History of trauma	3/5
** **Idiopathic	2/4
Length of follow-up*∗* (years)	5 ± 3.5 (2-9)

*∗*The values are given as the mean and standard deviation with the range in parentheses. ARCO: the Association Research Circulation Osseous.

**Table 2:** The VAS and Harris hip scores before and after ESWT.

		After ESWT (months)			
Variable	Before ESWT	3	6	12	24
VAS*∗*	6.7 ± 2.1 (3-9)	2.4 ± 2.2 (0-5)	1.3 ± 1.2 (0-4)	0.5 ± 0.4 (0-2)	0.8 ± 1.1 (0-3)
P-value		<0.001	<0.001	<0.001	<0.001
HSS*∗*	73.2 ± 9.8 (53-89)	88.7 ± 7.1 (62-100)	91.7 ± 7.8 (65-100)	97.2 ± 2.2 (72-100)	96.7 ± 3.1 (73-100)
P-value		<0.001	<0.001	<0.001	<0.001

*∗* The values are given as the mean and standard deviation with the range in parentheses. VAS: visual analog scale; ESWT: extracorporeal shock wave therapy; and HSS = Harris hip score.

**Table 3:** The overall clinical outcomes after ESWT.

Clinical outcome	ARCO grade-I (*n* = 5 hips)	ARCO grade-II (*n* = 28 hips)	Total series (*n* = 33 hips)
	no. (%)	no. (%)	no. (%)
Improved	4 (80%)	17 (60.7%)	21 (63.3%)
Unchanged	1 (20%)	4 (14.3%)	5 (15.15%)
Worse	0 (0%)	7 (25%)	7 (21.2%)
Total hip arthroplasty	0 (0%)	4 (14.3%)	4 (12.1%)

ESWT: extracorporeal shock wave therapy; ARCO: the Association Research Circulation Osseous.

**Table 4:** Changes on plain radiographs and MRI images before and after ESWT.

Variable	Before ESWT	After ESWT	P-value	
Size of ONFH*∗* (%)	59 ± 32 (5-76)	28 ± 16 (0-63)	0.235	
Bone marrow edema				
** **Grade 0	3	20	0.003	
** **Grade 1	9	9		
** **Grade 2	16	4		
** **Grade 3	3	0		
** **Grade 4	2	0		

*∗*The values, given as the mean and standard deviation with the range in parentheses, represent the percentage of the involved area of the femoral head. ESWT: extracorporeal shockwave therapy; ONFH = osteonecrosis of the femoral head.

## Data Availability

The data used to support the findings of this study are included within the article.
